# Association of the *p75^NTR^* Ser205Leu Polymorphism with Asymptomatic HTLV-1 Infection

**DOI:** 10.3390/v14061175

**Published:** 2022-05-28

**Authors:** Maria Alice Freitas Queiroz, Felipe Teixeira Lopes, Bruno José Sarmento Botelho, Maria Karoliny da Silva Torres, Ednelza da Graça Silva Amoras, Carlos A. da Costa, Maísa Silva Sousa, Ricardo Ishak, Antonio Carlos Rosário Vallinoto

**Affiliations:** 1 Laboratory of Virology, Institute of Biological Sciences, Federal University of Pará (UFPA), Belém 66075-110, Brazil; feliptlopes1@gmail.com (F.T.L.); sarmentobruno7@gmail.com (B.J.S.B.); karolinytorres15@gmail.com (M.K.d.S.T.); ednelza@hotmail.com (E.d.G.S.A.); rishak@ufpa.br (R.I.); vallinoto@ufpa.br (A.C.R.V.); 2 Graduate Program in Biology of Infectious and Parasitic Agents, Institute of Biological Sciences, Federal University of Pará (UFPA), Belém 66075-110, Brazil; 3 Laboratory of Cellular and Molecular Biology, Tropical Medicine Center, Federal University of Pará (UFPA), Belém 66055-240, Brazil; carauco@gmail.com (C.A.d.C.); maisaufpa@gmail.com (M.S.S.)

**Keywords:** HTLV-1, HAM, NGF, p75^NTR^, polymorphisms, proviral load, cytokines

## Abstract

Genetic variations in components of the immune response seem to be an important factor that contributes to the manifestation of symptoms of some diseases related to HTLV-1 infection. Nerve growth factor (NGF) and the p75 neurotrophin receptor (p75^NTR^) are related to the maintenance of neurons and the activation of the immune response. In this study, we evaluated the association of the *NGF* -198C/T, *NGF Ala35Val*, and *p75^NTR^ Ser205Leu* polymorphisms with HTLV-1 infection and plasma cytokine levels in 166 samples from individuals infected with HTLV-1 (59 symptomatic and 107 asymptomatic). The genotyping and quantification of the proviral load were performed by real-time PCR, and cytokine levels were measured by ELISA. The NGF -198C/T and NGF Ala35Val polymorphisms were not associated with HTLV-1 infection. The frequency of the *Ser/Leu* genotype of p75^NTR^ Ser205Leu was more frequent in the control group (*p* = 0.0385), and the *Ser/Leu* genotype and allele *Leu* were more frequent among the asymptomatic (*p* < 0.05), especially with respect to the HTLV-1-associated myelopathy (HAM) group (*p* < 0.05). The symptomatic showed a higher proviral load and higher TNF-α and IL-10 levels (*p* < 0.05). Asymptomatic carriers of the *Ser/Leu* genotype (*p* = 0.0797) had lower levels of proviral load and higher levels of TNF-α (*p* = 0.0507). Based on the results obtained, we conclude that the *p75^NTR^ Ser205Leu* polymorphism may be associated with reduced susceptibility to HTLV-1 infection, a lower risk of developing symptoms, including HAM, and better infection control.

## 1. Introduction

Human T-lymphotropic virus 1 (HTLV-1) is etiologically responsible for adult T-cell leukemia (ATL) and HTLV-1-associated myelopathy (HAM) and is associated with the development of other inflammatory diseases, such as dermatitis, rheumatic diseases, and uveitis [1,2,3]. Although it was isolated for the first time in 1979 [4], the factors that lead to the pathogenesis of diseases associated with HTLV-1 are still not well understood because only approximately 5% of people living with HTLV-1 (PLHTLV) develop some type of disease. Approximately 5 to 10 million people are infected with HTLV-1 worldwide [5]. Unfortunately, HTLV-1 infection, which mainly affects people with a low economic status, continues to be neglected, and little has been invested in research on the virus [6,7,8].

Changes in host anti-HTLV-1 immune responses seem to be an important condition that contributes to the manifestation of symptoms of some infection-related diseases and have been the focus of several studies in the search for biomarkers that can explain the evolution of infection and the development of associated diseases. Genetic variations in genes encoding cytokines, restriction factors, and proteins that activate apoptosis have been associated with the development of HAM and other inflammatory manifestations associated with HTLV-1 [9,10,11,12,13,14].

Nerve growth factor (NGF) is primarily related to neuron survival and the maintenance of neuronal structure [15], but it also plays an important role in the activation of the immune response and is involved in the proliferation of lymphocytes and monocytes as well as in the control of the synthesis and release of other cytokines [16]. The main receptors involved in the activation of NGF functions are the tropomyosin kinase A (TrkA) receptor and the p75 neurotrophin receptor (p75^NTR^), also called NGFR [17]. p75^NTR^ is a low-affinity receptor that promotes cell survival when it interacts with receptors of the tyrosine kinase (Trk) family [18]. In addition, the expression of p75^NTR^ has been described in several immune cells, and in the absence of TrkA, it can promote the activation of T lymphocyte effector responses [17].

Several single nucleotide polymorphisms (SNPs) have been described in the promoter (rs11102930) and coding (rs6325, rs6330, rs11466110, rs11466111) regions of the NGF gene [19,20,21,22]. In the p75^NTR^ gene, 12 SNP tags were identified, i.e., rs2537710, rs603769, rs614455, rs2537706, rs534561, rs2072445, rs2072446, rs7219709, rs734194, rs741071, rs741073, and rs2671641, which represent a total of 51 SNPs [23].

The NGF polymorphism rs11102930 corresponds to a substitution of cytosine with thymine at position -198 of the promoter region (*NGF* -198C/T), and it modifies the transcription factor binding site and possibly NGF expression [19]. The rs6330 SNP is located at position +273 of exon 3 of NGF and results in the substitution of cytosine with thymine, leading to a change in the amino acid alanine with valine at position 35 of the peptide (*Ala35Val*) and, consequently, a change in the structure of NGF [20]. The polymorphism rs2072446 in the p75^NTR^ gene is characterized by the substitution of cytosine with thymine at nucleotide 727 of exon 4, resulting in the replacement of the amino acid serine with leucine at position 205 of the peptide structure of the receptor (*Ser205Leu*), altering its functions [24].

These three polymorphisms stand out mainly because they have been associated with several diseases, including Alzheimer’s disease [25], multiple sclerosis, and a degenerative inflammatory neurological disease [20], as well as with the pathogenesis of infectious inflammatory disease [26]. Thus, the present study evaluated the association of the *NGF* -198C/T (rs11102930), *NGF Ala35Val* (rs6330), and *p75^NTR^ Ser205Leu* (rs2072446) polymorphisms with HTLV-1 infection, the presence of diseases associated with infection, proviral load, and plasma levels of the cytokines TNF-α, IFN-γ, and IL-10.

## 2. Materials and Methods

### 2.1. Characterization and Sample Collection

Blood samples were collected from 166 PLHTLV-1, i.e., 59 patients with a clinical diagnosis of inflammatory diseases (34 diagnosed with HAM, 17 with rheumatologic manifestations, 2 with dermatitis, 1 with uveitis, and 5 with more than 1 diagnosis) and 107 asymptomatic patients. Individuals of both sexes, older than 18 years, without treatment with glucocorticoids, and treated at the outpatient clinic of the Center for Tropical Medicine of the Federal University of Pará (NMT-UFPA) were included in the study.

A 10 mL sample of blood was collected by intravenous puncture using a vacuum collection system containing ethylenediaminetetraacetic acid (EDTA) as an anticoagulant. The samples were centrifuged and separated into plasma and leukocyte fractions. The leukocyte samples were used for the extraction of genomic DNA to genotype the *NGF* -198C/T, *NGF Ala35Val*, and *NGFR Ser205Leu* SNPs and quantify the proviral load. Plasma samples were used to measure the concentrations of TNF-α, IFN-γ, and IL-10.

For comparison, blood samples were collected from 200 individuals negative for HTLV-1/2, HIV-1/2, HCV, and HBV who were older than 18 years and without autoimmune disease. These samples served as the control group and were used to compare polymorphism frequencies; they were representative of the same population group that constituted the PLHTLV group. Therefore, there were no interethnic differences.

### 2.2. DNA Extraction

DNA was extracted from whole blood leukocytes using a Puregene™ kit (QIAGEN, Hilden, Germany) according to the manufacturer’s protocol (briefly, cell lysis, protein precipitation, DNA precipitation, and hydration). After extraction, the DNA obtained was quantified by spectrophotometry in a BioDrop™ instrument (Bio-Rad, Hercules, CA, USA) following the protocol recommended by the manufacturer.

### 2.3. Genotyping of NGF -198C/T, NGF Ala35Val, and p75^NTR^ Ser205Leu

The identification of the polymorphism genotypes was performed by real-time PCR using a StepOnePLUS™ Real-Time PCR System (Thermo Fisher, Carlsbad, CA, USA). The reactions were conducted using commercially obtained TaqMan™ assays (*NGF* -198C/T (C__26680904_10), *NGF Ala35Val* (C__2525309_10) and *p75^NTR^ Ser205Leu* (C__15870920_10)) and contained primers and probes specific for the amplification of the target sequence (Thermo Fisher, Carlsbad, CA, USA). The reaction consisted of 1× MasterMix, H_2_O, 20× assay C_11537906_20, and 50 ng of DNA, and the cycling conditions were as follows: 10 min at 95 °C and 40 cycles of 15 s at 95 °C and 1 min at 60 °C.

### 2.4. Quantification of HTLV-1 Proviral Load

Proviral load was quantified by qPCR using 3 target sequences, which were synthesized using the TaqMan^®^ system (Life Technologies, Foster City, CA, USA) according to a previously described protocol [27], which begins with the collection of 5 mL of whole blood for DNA extraction from leukocytes, followed by relative quantification using real-time PCR. The result obtained was adjusted to obtain an absolute proviral quantification considering the leukocyte counts per mm^3^; the final result is expressed as proviral DNA copies/mm^3^.

### 2.5. Plasma Cytokine Level

Plasma TNF-α, IFN-γ, and IL-10 was quantified using the Ready-SET-Go^®^ enzyme-linked immunosorbent assay (ELISA) (eBioscience, San Diego, CA, USA), which uses specific monoclonal antibodies to detect each of the cytokines. The test was performed according to the manufacturer’s instructions.

### 2.6. Statistical Analysis

The genotypic and allelic frequencies of the polymorphisms were estimated by direct counting, and the significance of the differences between the studied groups was calculated using the χ2 (Chi-square) test, Fisher’s exact test, and G test. Hardy–Weinberg equilibrium was calculated to evaluate whether the distributions of observed genotypic frequencies were in accordance with expectations. The distribution of proviral load and cytokine levels were evaluated using the Shapiro–Wilk test. The test indicated that the data did not follow a normal distribution; therefore, the analyses used non-parametric tests. The dosage of proviral load was evaluated using the Mann–Whitney and Kruskal–Wallis tests. The concentration of cytokines was analyzed by the Mann–Whitney test. All tests were performed using BioEstat 5.3 and GraphPad Prism 5.0 software, and *p* ≤ 0.05 was considered significant.

## 3. Results

The distribution of the genotypic frequencies of the *NGF* -198C/T, *NGF Ala35Val*, and *p75^NTR^ Ser205Leu* polymorphisms in all evaluated groups were in Hardy–Weinberg equilibrium (*p* > 0.05). The frequencies of the genotypes and alleles for the *NGF* -198C/T and *NGF Ala35Val* polymorphisms were not significantly different between the group of individuals infected with HTLV-1 and the control group; however, for the *p75^NTR^ Ser205Leu* polymorphism, the frequency of the Ser/Leu genotype was significantly higher among individuals in the control group (*p* = 0.0385). The Leu/Leu genotype was not present in any of the groups (Table 1).

The comparison of the genotypic and allelic frequencies of the polymorphisms between individuals with and without symptoms indicated that there were no significant differences in relation to the *NGF* -198C/T and *NGF Ala35Val* polymorphisms. In contrast, for the *p75^NTR^ Ser205Leu* polymorphism, there was a higher frequency of the *Ser/Leu* genotype (*p* = 0.0082) and the *Leu* allele (*p* = 0.0289) in the group of asymptomatic individuals than in the group with clinical symptoms, in which the presence of the *Ser/Leu* genotype was not observed (Table 2).

Among the asymptomatic and HAM groups (Table 3), there was also no significant difference in the frequencies of genotypes and alleles for *NGF* -198C/T and *NGF Ala35Val*. For the *p75^NTR^ Ser205Leu* polymorphism, the presence of polymorphic genotypes was not found in the HAM group; therefore, the frequency of the heterozygous genotype and polymorphic allele in the asymptomatic group was significantly higher (*p* = 0.0381 and *p* = 0.0289, respectively).

**Figure 1:**
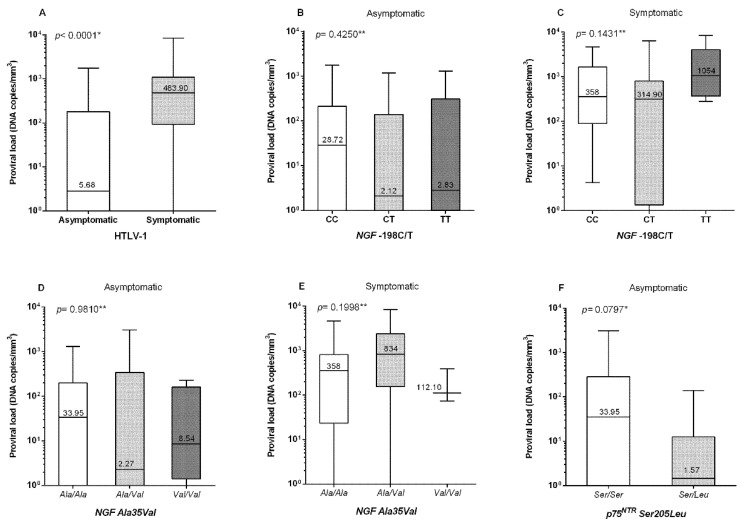
HTLV-1 proviral loads for (**A**) the asymptomatic and symptomatic groups; for individuals with the *NGF* -198C/T genotypes in the (**B**) asymptomatic and (**C**) symptomatic groups; for individuals with the *NGF Ala35Val* genotype in the (**D**) asymptomatic and (**E**) symptomatic groups; and for individuals with the *p75^NTR^ Ser205Leu* genotype in the (**F**) asymptomatic group. * Mann–Whitney test; ** Kruskal–Wallis test; *p*-value < 0.05: statistically significant; *p*-value > 0.05: not statistically significant; *p*-value < 0.01: highly statistically significant.

The HTLV-1 proviral load levels were higher in individuals with symptoms than in asymptomatic individuals (*p* < 0.0001; Figure 1A). There was no significant difference in the proviral load levels between the *NGF* -198C/T and *NGF Ala35Val* polymorphism genotypes in the asymptomatic group or in the group of patients with symptoms (Figure 1B–E). However, for the *p75^NTR^ Ser205Leu* polymorphism, individuals with the *Ser/Leu* genotype had lower proviral load levels, with a *p*-value close to significance (*p* = 0.0797) for the asymptomatic group (Figure 1F).

**Figure 2:**
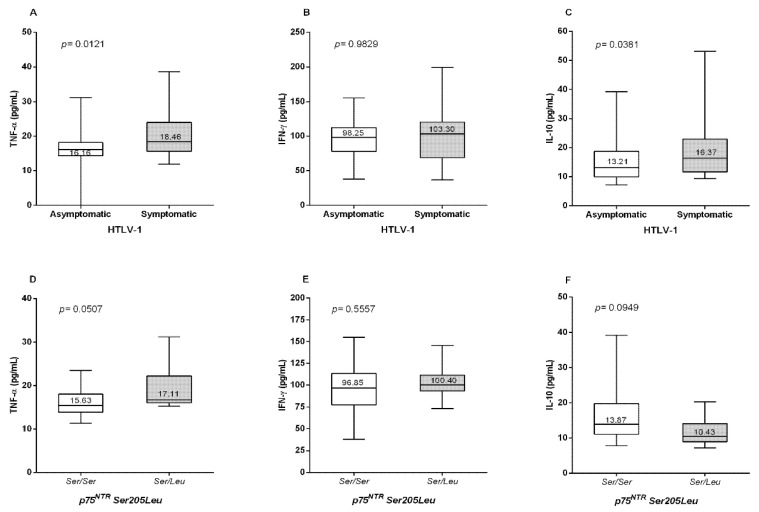
Comparison of plasma levels of (**A**) TNF-α, (**B**) IFN-γ, and (**C**) IL-10 between the asymptomatic and symptomatic groups; (**D**) TNF-α, (**E**) IFN-γ, and (**F**) IL-10 levels for individuals with the *p75^NTR^ Ser205Leu* genotype. Mann–Whitney test; *p*-value < 0.05: statistically significant; *p*-value > 0.05: not statistically significant; *p*-value < 0.01: highly statistically significant.

The levels of the TNF-α, IFN-γ, and IL-10 were evaluated in symptomatic and asymptomatic individuals and compared between the *p75^NTR^ Ser205Leu* genotypes of the asymptomatic group (which indicated significant differences in frequencies between the groups evaluated and variations in viral load levels). The results of the cytokine level evaluation indicated that the symptomatic group had significantly higher levels of TNF-α (*p* = 0.0121; Figure 2A) and IL-10 (*p* = 0.0381; Figure 2C). The levels of IFN-γ were not significantly different between the groups (Figure 2B). Regarding the *p75^NTR^ Ser205Leu* polymorphism, *Ser/Leu* genotype carriers had higher levels of TNF-α (*p* = 0.0507; Figure 2D) and IFN-γ, but the differences were not statistically significant (Figure 2E); the carriers of this genotype had lower levels of IL-10 (Figure 2F).

## 4. Discussion

Several efforts have been made to better understand the dynamics of HTLV-1 infection and the development of different symptoms in certain people, as well as characteristics inherent to the virus and variations in the host’s immune response to infection [28,29,30,31]. Genetic variations in important elements of the immune response have been investigated as potential biomarkers for the development of symptoms related to HTLV-1 infection [12].

This study investigated the association of the *NGF* -198C/T, *NGF Ala35Val*, and *p75^NTR^ Ser205Leu* polymorphisms with HTLV-1 infection and HAM. The frequencies of polymorphisms in the NGF gene were not associated with HTLV-1 infection or with HAM. NGF and the p75^NTR^ receptor are related to two main functions: maintaining the survival and structure of neurons and contributing to the activation of the immune response [15,16].

Regarding the maintenance of neurons, it is assumed that variations in NGF levels could be related to one of the main diseases caused by HTLV-1, i.e., HAM, which is a slow-progressing inflammatory neurological disease characterized by axonal degeneration and demyelination, mainly in the corticospinal tract (group of axons that extend from the brain to the spinal cord) [32]. However, the investigated polymorphisms related to variations in NGF expression levels and structure were not associated with the presence of symptoms of diseases associated with HTLV-1, including HAM. Albrechet et al. (2006) evaluated the levels of NGF in the spinal cord of patients with HAM but found no differences in the levels of NGF between patients with HAM and individuals without infection, and they suggested that the levels of NGF might not be associated with HAM because NGF levels are not able to maintain the axon structure in cases of selective injury in slow processes [33]. The evaluation of NGF levels among patients with HAM and a control group of asymptomatic individuals with HTLV-1 infection may provide a better understanding of the role of NGF in the development of HAM.

The wild-type *C allele for the *NGF* -198C/T polymorphism has been considered protective against multiple sclerosis in males, who show higher levels of NGF expression [20], while the Val allele for the *NGF Ala35Val* polymorphism represents a risk factor for the development of Alzheimer’s disease [21]. In this sense, it is possible that the genetic polymorphisms *NGF* -198C/T and *NGF Ala35Val* do not influence the levels and functions of NGF in neurological diseases of infectious etiology; therefore, NGF would not contribute to determining the course of infection by HTLV-1 and the development of HAM.

In contrast, for polymorphisms in the p75^NTR^ receptor gene, the frequency of the polymorphic heterozygous genotype (Ser/Leu) and the polymorphic allele (Leu) were associated with asymptomatic infection. A similar result was observed by Cozza et al. (2008), who observed that the polymorphic allele had a protective effect against Alzheimer’s disease [21]. This is the first study that evaluated polymorphisms in the *NGF* gene (-198C/T and Ala35Val) and *p75^NRT^*(Ser205Leu) in HTLV-1 infection. Based on the analysis of the frequencies of the *p75^NRT^* Ser205Leu polymorphism between control individuals and individuals with HTLV-1, a greater association of infection with the wild type (Ser205Ser) genotype is suggested.

NGF has neuroprotective effects and can influence neural responses to injury in cell types that exhibit NGF receptors, such as nociceptive sensory neurons and motor neurons [34]. Trk is a high-affinity receptor for NGF, and the p75^NTR^ receptor is a low-affinity receptor for NGF [17]. However, high-affinity binding by NGF requires coexpression and binding to both the low-affinity NGF receptor (p75^NTR^) and the Trk receptor [35]. The coexpression and binding of both receptors increases the NGF association rate 25-fold, producing a binding site of greater affinity, which also promotes an increase in the internalization rate. High-affinity binding and internalization are prerequisites for the biological activities of NGF [36].

The results of the present study may indicate that the *p75^NTR^ Ser205Leu* polymorphism promotes an improvement in the functions of the p75^NTR^ receptor and optimizes Trk-NGF-p75^NTR^ binding; this would lead to better NGF activity, which would contribute to avoiding the development of HAM in some infected patients. However, this genetic alteration would be only one factor among other factors that could contribute to preventing the development of HAM. This hypothesis needs to be tested in studies with a greater number of patients with HAM and that evaluate the expression of NGF and p75^NTR^ in asymptomatic individuals and patients with HAM.

The involvement of NGF and p75^NTR^ in the immune response was initially observed in inflammatory and autoimmune diseases, inducing the activation of immune cells and promoting increased cytokine production. High levels of NGF were found at sites of inflammation. In addition, inflammatory cytokines can also induce NGF synthesis in a wide variety of cells, including neuronal, epithelial, endothelial, connective, and muscle cells [17].

In the present study, higher proviral loads and levels of TNF-α and IL-10 were observed in the group of patients with symptoms of diseases associated with HTLV-1 infection, suggesting that these patients had greater viral replication and inflammatory responses. However, there was no association of NGF gene polymorphisms with proviral loads and cytokine levels found between patients with and without symptoms. However, the polymorphism located in the NGF promoter region was previously shown to be associated with infectious inflammatory processes. Pereira et al. (2020) evaluated the *NGF* -198C/T polymorphism in patients with chronic viral hepatitis and observed that the TT polymorphic genotype frequency was higher in patients with higher degrees of inflammation and fibrosis [26] and that NGF expression levels were higher in patients with this histological profile [37].

Regarding the *p75^NTR^* Ser205Leu polymorphism, only asymptomatic individuals showed a frequency of the polymorphic genotype, with no association between the polymorphism and the development of symptoms associated with HTLV-1 infection, including HAM/TSP. Furthermore, the *p75^NTR^ Ser205Leu* polymorphism, asymptomatic individuals carrying the heterozygous polymorphic genotype had lower proviral loads, higher TNF-α levels, and lower IL-10 levels. Patients with viral hepatitis, carriers of the polymorphic genotypes for *p75^NTR^ Ser205Leu*, had lower levels of inflammatory activity [26]. NGF activity increases the production of proinflammatory cytokines, including that of IL-β and TNF-α [38,39].

A previous study showed that the p75^NTR^ receptor can interact with NGF and promote the secretion of IL-12 and TNF-α, suggesting that the receptor contributes to the induction of a Th1 response [40]. Thus, the results of the present study regarding higher levels of TNF-α in asymptomatic individuals carrying the Ser/Leu genotype seem to corroborate the possible association of the *p75^NTR^ Ser205Leu* polymorphism with improved receptor function, promoting a more effective interaction with Trk and NGF and intensifying neutrophin function, with the consequent induction of Th1 cytokine production. The regulation of NGF receptor functions during the differentiation and response of immune cells suggests a differential need for NGF depending on its state of maturity and functional activity [17].

The results of the evaluation of the presence of the *p75^NTR^ Ser205Leu* polymorphism, viral load, and cytokine levels in asymptomatic individuals suggested that the polymorphism may induce higher levels of TNF-α, which would contribute to the control of infection, leading to a lower viral load without promoting a marked inflammatory response (individuals with the *Ser/Leu* genotype had lower levels of IL-10); this is different from what was observed in patients with symptoms related to HTLV-1 infection, who had higher levels of TNF-α and IL-10 and a high proviral load.

Despite the important results observed in the present study, the need of evaluating epidemiological data related to HTLV-1 infection, such as the transmission route of the virus, is highlighted. Overall, the HTLV-1 proviral load value among HTLV-1 carriers who were infected by breastfeeding was reported as high [41]. As not all individuals involved in the study were aware of this information, this was a limiting factor of our study, requiring further investigations on the relationship of polymorphisms, proviral load, and the transmission route of HTLV-1.

## 5. Conclusions

In summary, the results of this study showed that important polymorphisms located in the NGF gene (*NGF* -198C/T and *NGF Ala35Val*) were not associated with HTLV-1 infection. In contrast, the *p75^NTR^ Ser205Leu* polymorphism, which is located in a low-affinity receptor, was associated with reduced susceptibility to HTLV-1 infection, a lower risk of developing symptoms, including HAM, and better infection control.

## Figures and Tables

**Table 1:** Distribution of the genotypic and allelic frequencies of *NGF* -198C/T, *NFG Ala35Val*, and *p75^NTR^ Ser205Leu* between the group of individuals infected with HTLV-1 and the control group.

Genotypes and Alleles	HTLV-1 *n* = 166 *n* (%)	Control *n* = 200 *n* (%)	*p*
*NGF* -198C/T rs11102930			
CC	53 (31.9)	67 (33.5)	0.9358 *
CT	83 (50.0)	99 (49.5)	
TT	30 (18.1)	34 (17.0)	
* C	0.57	0.58	1.0000 **
* T	0.43	0.42	
*NGF Ala*35*Val* rs6330			
*Ala/Ala*	77 (46.4)	90 (45.0)	0.2389 *
*Ala/Val*	79 (47.6)	88 (44.0)	
*Val/Val*	10 (6.0)	22 (11.0)	
*Ala*	0.70	0.67	0.6519 **
*Val*	0.30	0.33	
*p75^NTR^ Ser*205*Leu* rs2072446			
*Ser/Ser*	155 (93.4)	173 (86.5)	0.0385 **
*Ser/Leu*	11 (6.6)	27 (13.5)	
*Ser*	0.97	0.93	0.3311 **
*Leu*	0.03	0.07	

*n* = Number of individuals; * Chi-square test; ** Fisher’s exact test.

**Table 2:** Distribution of genotypic and allelic frequencies of *NGF* -198C/T, *NFG* Ala35Val and *p75^NTR^* Ser205Leu among asymptomatic individuals and patients with symptoms associated with HTLV-1 infection.

Genotypes and Alleles	Asymptomatic *n* = 107 *n* (%)	Symptomatic *n* = 59 *n* (%)	*p*
*NGF* -198C/T rs11102930			
CC	31 (29.0)	22 (37.3)	0.3415 *
CT	58 (54.2)	25 (42.4)	
TT	18 (16.8)	12 (20.3)	
* C	0.56	0.58	0.8895 **
* T	0.44	0.42	
*NGF Ala*35*Val* rs6330			
*Ala/Ala*	53 (49.6)	24 (40.7)	0.4550 ***
*Ala/Val*	47 (43.9)	32 (54.2)	
*Val/Val*	7 (6.5)	3 (5.1)	
*Ala*	0.71	0.68	0.649 **
*Val*	0.29	0.32	
*p75^NTR^ Ser*205*Leu* rs2072446			
*Ser/Ser*	96 (89.7)	59 (100)	0.0082 **
*Ser/Leu*	11 (10.3)	0	
*Ser*	0.94	1	0.0289 **
*Leu*	0.06	0	

*n* = Number of individuals; * Chi-square test; ** Fisher’s exact test; *** G Test.

**Table 3:** Distribution of genotypic and allelic frequencies of *NGF* -198C/T, *NFG Ala35Val* and *p75^NTR^ Ser205Leu* among individuals infected with HTLV-1 without clinical symptoms and with HAM.

Genotypes and Alleles	Asymptomatic *n* = 107 *n* (%)	HAM *n* = 38 *n* (%)	*p*
*NGF* -198C/T rs11102930			
CC	31 (29.0)	13 (34.2)	0.8332 *
CT	58 (54.2)	19 (50.0)	
TT	18 (16.8)	6 (15.8)	
* C	0.56	0.59	0.7749 **
* T	0.44	0.41	
*NGF Ala*35*Val* rs6330			
*Ala/Ala*	53 (49.6)	16 (42.1)	0.3899 ***
*Ala/Val*	47 (43.9)	21 (55.3)	
*Val/Val*	7 (6.5)	1 (2.6)	
*Ala*	0.71	0.69	0.8775 **
*Val*	0.29	0.31	
*p75^NTR^ Ser*205*Leu* rs2072446			
*Ser/Ser*	96 (89.7)	38 (100)	0.0381 **
*Ser/Leu*	11 (10.3)	0	
*Ser*	0.94	1	0.0289 **
*Leu*	0.06	0	

*n* = Number of individuals; * Chi-square test; ** Fisher’s exact test; *** G Test.

## Data Availability

The data analyzed in this study are included within the paper.
